# Screening of the pelvic organ prolapse without a physical examination; (a community based study)

**DOI:** 10.1186/1472-6874-11-48

**Published:** 2011-11-15

**Authors:** Fahimeh Ramezani Tehrani, Somayeh Hashemi, Masoumeh Simbar, Niloofar Shiva

**Affiliations:** 1 Reproductive Research Center, Research Institute for Endocrine Sciences, Shahid Beheshti University of Medical Sciences, (Yaman), Tehran, (1985717413), Iran

## Abstract

**Background:**

Pelvic organ prolapse (POP) is a silent disorder with a huge impact on women's quality of life. There is limited data from community-based studies conducted to determine the prevalence of POP as its assessment needs a pelvic examination. We aimed to develop a simple screening inventory for identification of pelvic organ prolapse and then evaluate its sensitivity and specificity.

**Methods:**

This study had two phases. In the first phase in order to develop a simple inventory for assessment of POP, the Pelvic Floor Disorder Inventory (PFDI) was completed for a convenience sample of 200 women, aged 18-45 years, referred for annual gynecologic examination, and their pelvic organ prolapse was assessed using the standard protocol. The most sensitive and specific questions were selected as pelvic organ prolapse simple screening inventory (POPSSI). In the second phase, using a stratified multistage probability cluster sampling method, the sensitivity and specificity of the POPSSI was investigated in a non selected sample of 954 women recruited from among reproductive aged women living in four randomly selected provinces of Iran.

**Results:**

The sensitivity and specificity of POPSSI for identification of pelvic organ prolapse in the general population were 45.5 and 87.4% respectively; these values were 96.7 and 20% among those women who were aware of their pelvic dysfunction.

**Conclusion:**

Community based screening studies on pelvic organ prolapse could be facilitated by using the POPSSI, the sensitivity of which would be enhanced through conducting of public awareness programs.

## Background

Pelvic organ prolapse (POP), a common disorder resulting from relaxation of the pelvic floor muscles [1,2], is estimated to have a prevalence of 30-50% among women, aged 50 and over [3]. Although mortality resulting from POP is not significant[4], it has a huge impact on the daily activities of women afflicted by this condition, often disrupting and decreasing their quality of life[5]. Pelvic organ prolapse and its complications impose a considerable economic burden on the person and it has been estimated that about 11% of women undergo surgery for POP before the age of 79 with 29.2% requiring repeated surgery [6-8]. Subramanian et al noted that the cost of surgery for pelvic organ prolapse in 2005 was 144,236,557 euro, 83,067,825 euro, and 81,030,907 euro in Germany, France, and England, respectively[9]

There are few community-based studies conducted to determine the prevalence of pelvic organ prolapse[10]. Requirement of a pelvic examination is a major hindrance for such studies and needs considerable time, resources and costs. Moreover the embarrassment and discomfort associated with clinical examination is also a significant restriction for the participants. Various screening questions have been demonstrated for assessment of POP, but their feasibility was restricted due to the large number of required questions[11-13]. Following efforts to modify the questionnaires and simplify them, the World Health Organization declared four main questions that have been able to correctly identify 80-90% of POP patients[14]. On the other hand, Tegerstedt et al presented a screening method based on five questions[15]. Since applying these modified questions had different results in various countries[16-20], it seems that socio-cultural factors may restrict its application in various societies. We aimed to develop a simple screening inventory for identification of pelvic organ prolapse and to evaluate its sensitivity and specificity in a general population of Iranian women.

## Methods

This descriptive study was performed in 2 phases;

### First phase

(Developing a pelvic organ prolapse simple screening inventory (POPSSI) based on Pelvic Floor Disorder Inventory (PFDI).)

Initially, the PFDI [10] was selected as the preliminary tool for POP screening. This inventory is a questionnaire consisted of 20 questions on the symptoms of POP. PFDI was translated to Persian by the researcher and then translated back to English by an official English language translator, who is an expert in translating English medical texts. Then, the content validity of the Persian version of PFDI was assessed by 15 gynecologists and reproductive health experts. The reliability of this questionnaire was also assessed by the test- retest method, and was confirmed by r = 0.91.

The standard protocol that was described in detail by Bump et al was used for diagnosis of POP[21]. According to this protocol the subjects were assigned a POPQ stage as follows : stage I, leading edge of the prolapse is >1 cm above the hymen; stage II, leading edge of the prolapse is ≤1 cm proximal or distal to the plane of the hymen; stage III, leading edge of the prolapse is >1 cm below the plane of the hymen but protrudes no further than 2 cm less than the total vaginal length, and stage IV, essentially complete eversion of the total lower genital tract[21]. The content validity of this checklist was also assessed by the above mentioned experts as well. The reliability of this checklist, assessed using the inter-rater reliability method, was confirmed by r = 0.85.

A convenience sample of 200 reproductive aged women, referred for annual gynecologic examinations, filled out the Persian version of PFDI and underwent pelvic examination using the standard protocol for assessment of POP. Two different potential cut off points were considered for POP diagnosis: 1- Observing at least one form of POP with intensity to ≥1. 2: Observing at least one form of POP with intensity to ≥2.

Subsequently the area under the receiver operating characteristics [22] curve was calculated to assess the ability of each question of the PFDI. Using the ROC curve, the most valid questions were identified based on optimal values for sensitivity and specificity as the ones that keep (1 - sensitivity)^2 ^+ (1 - specificity)^2 ^at minimum [23]. As a result the POPSSI consisted of these 4 questions: 1) Urinary incontinence following laughing, sneezing or coughing; 2) Urinary urgency; 3) Feeling pain during defecation; 4) Feeling or seeing bulge in vagina. The content validity of the POPSSI was assessed by 15 gynecologists and reproductive health experts. The reliability of the questionnaire was assessed using test-retest and inter related method. both confirm by r = 0.91 and r = 0.85 respectively.

### Second phase

(Assessment of POPSSI among general population.)

In the second phase of this study, 1200 women, aged 18-45 years, were recruited from among reproductive aged women living in an urban area of four randomly selected provinces in different geographic regions, i.e. Ghazvin(Central), Kermanshah[24], Golestan (North) and Hormozgan (South). A stratified, multistage probability cluster sampling method, with a probability in proportion to size procedure, was used, each cluster comprising of seven households. The choice of seven households for the cluster size was based on the one-day performance capacity of the data collection group. The frame for the selection of the sampling units was based on the Iranian household lists available in the Health Department and the cluster was selected systematically. The proportion of required samples in each province was calculated based on the total number of women aged 18-45 living in the urban areas of each of these provinces.

During face to face interviews a standard questionnaire including POPSSI questionnaire and information on socio-demographic and reproductive was completed by trained midwives, and all participants underwent vaginal examinations using standard protocol under supervision of one gynecologist for each province. Pregnant or postmenopausal women and those with a history of hysterectomy, ovarectomy or any surgery because of pelvic organ prolapse were excluded. Sensitivity, specificity, positive and negative predictive value of POPSSI were calculated using the same protocol mentioned for first phase.

Data were analyzed using SPSS 15 statistical software (SPSS Inc., Chicago, IL).

The national ethical review board approved the study and informed consent was obtained from all subjects.

## Results

### First phase

**Figure 1:**
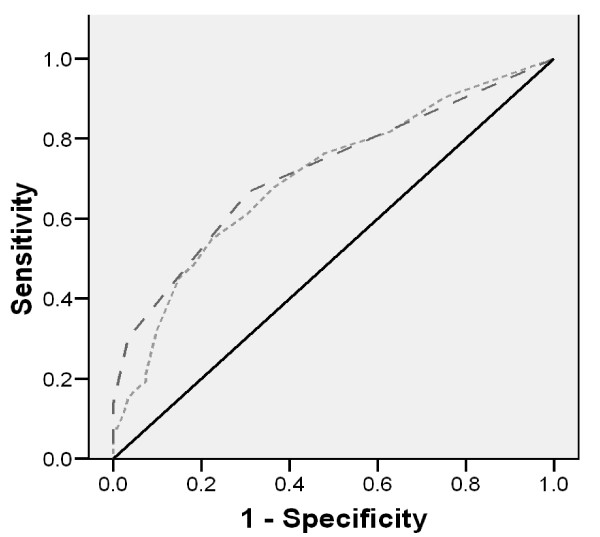
**Receiver operating characteristics curve for using PFDI and POPSSI to predict pelvic organ prolapse**.

The mean age of participants was 34.2 ± 9.4, and a majority of them (80.4%) had educational levels of high school or above. Of participants, 60.8% had the previous history of at least one pregnancy and the mean and standard deviation of their parity was 1.4 ± 1.5. In 46.1% of the women, at least one type of POP, with intensity ≥1 was observed. The sensitivity and specificity of the PFDI were 81.8% and 54.8%, respectively. ROC analysis determined that the four questions selected, had approximately the same sensitivity (84.7%) and specificity (47.4%) as the total 20 questions of the PFDI(Figure 1). Furthermore the question on urinary incontinence following laughing, sneezing or coughing was the most relevant question with a sensitivity of 35.4% and a specificity of 91.9%.

Applying the second approach (diagnosis of prolapse with intensity ≥2) for POP identification did not improve the ability of PFDI for correct identification of women with or without POP (sensitivity 92.4% and specificity 42.9%), which is why the 1^st ^approach was selected for development of POPSSI.

### Second phase

In the second phase of this study (POPSSI external validation), of the 1200 women who met our inclusion criteria, 945 completed the study procedure. The basic and reproductive characteristics of the study subjects are presented in Table 1. At least one type of POP with an intensity ≥1 was observed in 39.5% participants (24.5% grade I, 12.6% grade II, 2.2% grade III and 0.2% grade IV). The screening test characteristics of the POPSSI are presented in table 2 (sensitivity = 45.5%, specificity = 87.4%).

**Table 1:** Basic and reproductive characteristics of study participants in the 2^nd ^phase of the study (n = 945)

	Mean ± Standard Deviation
**Age(years)**	34.3 ± 6.9
**Age at marriage (years)**	19.9 ± 3.3
**Education(years)**	8.5 ± 4.3
**Body mass index(kg/m2)**	27.7 ± 4.6
**Gravidity**	2.8 ± 1.5
**Parity**	2.4 ± 1.4
**Number of vaginal deliveries**	2.0 ± 1.6
**Number of abortions**	0.3 ± 0.6

**Table 2:** Screening characteristics of each question of the pelvic organ prolapse screening inventory (POPSSI) in the 2^nd ^phase of the study (n = 945)

	Sensitivity (%)	Specificity (%)	Positive predictive value (%)	Negative predictive value (%)	False negative (%)	False positive (%)
**Urinary incontinence following laughing, sneezing or coughing**	40.7	88	71.4	66.7	59.2	**12**
**Urinary urgency**	21.3	88.4	57.6	60.2	78.6	**11.6**
**Feeling pain during defecation**	3.9	97.1	50	57.7	96.1	**2.8**
**Feeling or seeing bulge in vagina**.	9.8	99	88.3	59.7	90.2	**0.9**
*POPSSI	45.5	87.4	72.7	68	54	**12.5**

*all of these four questions

Stratified analysis was carried out by splitting the data into two groups of women with or without previous awareness on their POP condition, the sensitivity and specificity of POPSSI for identification of each of this stratum are presented in Tables 3 and 4. Results demonstrated that POPSSI had a higher predictive value in women who already were aware of their POP condition, sensitivity 96.7% and specificity of 20.0%, in comparison to 39.3 and 89.1%, in women hither unaware of their POP status.

**Table 3:** Screening characteristics of pelvic organ prolapse simple screening inventory (POPSSI) among women who have already been aware about their pelvic organ prolapse situation.

		**POP based on pelvic examination**			
		
		**presence**		**absence**	
**POP based on POPSSI**	Yes	88		24	
	No	3		6	
Sensitivity (%)	Specificity (%)	Positive predictive value (%)	Negative predictive value (%)	False negative (%)	False positive (%)
96.7	20	78	66	34	22

**Table 4:** Screening characteristics of pelvic organ prolapse simple screening inventory (POPSSI) among women who had not already been aware of their pelvic organ prolapse status.

		**POP based on pelvic examination**			
		
**POP based on POPSSI**		**Presence**		**absence**	
	Yes	116		54	
	No	179		440	
Sensitivity (%**)**	Specificity (%)	Positive predictive value (%)	Negative predictive value (%)	False negative (%)	False positive (%)
39.3	89.1	68	71	29	32

## Discussion

The present study demonstrated that POPSSI with 45.5% sensitivity and 87.4% specificity could correctly identify the POP condition of about 50% of women in the general population. We found that the predictive value of POPSSI was enhanced for those women who were previously aware of their POP condition (sensitivity = 96.7%). Based on our results the simple question for "Urinary incontinence following laughing, sneezing or coughing'' had the best validity for prediction of pelvic organ prolapse.

Although several screening tools have been introduced to identify women suffering from pelvic organ prolapse, there is no consensus on a simple and accurate tool, yet. The World Health Organization (WHO) suggested a questionnaire with the four following questions for assessment of POP [14]; 1. Do you have feeling of vaginal bulging? 2. Do you have feeling of heaviness? 3. Do you have feeling of discomfort defecation? 4. Do you need manipulation for stool or urine discharge? WHO asserted this simple questionnaire was been able to appropriately assess the POP situation of more than 80% of women. However studies based on WHO suggestion yielded different results [16,20]. Tegerstedt et al provided a screening model, including 5 items with a sensitivity and specificity of 92.5% and 94.5% respectively for identifying prolapse in women referred to urogynecology clinics; however its sensitivity declined to 66.5% in surveys conducted on general populations. The single most valid item of Tegerstedt's questionnaire was vaginal bulging [15]. Lukacz et al found that the item of "sensation of something falling out of the vagina" had the best validity for prediction of pelvic organ prolapse [17]. In Tan et al's study " vaginal bulging" could correctly identify the POP condition in 80% of women and the lack of this symptom was observed in 70% of women without POP[25]; this item was one of our four selected questions.

The differences in results reported between these studies and those of ours can be explained by the different in symptoms' explored; cultural diversity [26] and various races [27]. Furthermore the burden of pelvic organ relaxation can be influenced by the "culture of silence" surrounding this condition, in particular, in countries with conservative backgrounds and because women who have had pregnancy consider this condition to be normal.

Also the characteristics of study participants could affect the validity of each screening test; as a result the sensitivity of the POPSSI screening test can be increased whenever it is applied for a group of women attending urogenital clinics in comparison to its application for women without any specific urogenital symptoms. On the other hand, the specificity of each POP screening test is enhanced if it is applied among ordinary women rather than those presenting to urogenital clinics[10]. Therefore we have done subgroup analysis of data obtained using the POPSSI questionnaire for both women who were previously aware of their POP condition and those unaware as well, to avoid biased results. Our findings indicated that the predictive value of POPSSI was significantly improved when the women were already aware of their POP condition. It seems that community awareness programs could possibly improve the ability of this simple inventory (POPSSI) for assessment of pelvic organ relaxation. The primary health care system in Iran with coverage of more than 90% [28] enables us to use this simple POPSSI questionnaire for identification of either those women suffering from POP that may be benefit from simple intervention or those who need to be referred to the second level of health care system for more specific interventions.

Our results were not influenced by menopausal status, as our study subjects were selected from among non menopausal women; it has however been shown that the clinical manifestations of POP are highly affected by menopause status; urinary incontinence and frequency may develop after menopause as a result of atrophic vaginitis and are not related to POP[29].

The main strength of the present study is its methodology, as it is a community based prevalence study, carried out on an ethnically homogenous population, with an appropriate response rate of about 90%. One of our study's limitations is not using the advanced diagnostic tools for classification of pelvic organ relaxation; however it has been shown that the standard protocol used for POP diagnosis in our study, has a acceptable diagnostic capability in comparison to those advanced methods[30]. We excluded menopausal women therefore our results is not applicable for these women.

## Conclusion

Our simple screening inventory for pelvic organ prolapse could facilitate community based studies by eliminating the pelvic examination; the community awareness program on this disorder may be considered as a perquisite and could enhance its predictive value. Screening of pelvic organ relaxation at the community level is highly recommended as its mild form can be easily treated using simple interventions.

## List of abbreviations

(POP): Pelvic Organ Prolapse; (PFDI): Pelvic Floor Disorder Inventory; (POPSSI): Pelvic Organ Prolapse Simple Screening Inventory; (ROC): Receiver Operating Characteristics; (WHO): World Health Organization

## Competing interests

The authors declare that they have no competing interests.

## Authors' contributions

FRT contributed to study design, execution, analysis, manuscript drafting and critical discussion. SH also contributed to analysis, manuscript drafting and critical discussion. MS contributed to study design, manuscript drafting and critical discussion. NS drafted the manuscript and contributed to language editing. All authors read and approved the final manuscript.

## Authors' information

Fahimeh Ramezani Tehrani, MD, Gynecologist, Professor, Reproductive Endocrinology Research Center, Research Institute for Endocrine Sciences, Shahid Beheshti University of Medical Sciences

Somayeh Hashemi, MS, Midwife, Researcher, Reproductive Endocrinology Research Center, Research Institute for Endocrine Sciences, Shahid Beheshti University of Medical Sciences

Masoumeh Simbar, PhD Reproductive Health, Associate Professor, Reproductive Endocrinology Research Center, Research Institute for Endocrine Sciences, Shahid Beheshti University of Medical Sciences

Niloofar Shiva, PhD Health and Communication, Research Institute for Endocrine Sciences, Shahid Beheshti University of Medical Sciences, Tehran, Iran

## Pre-publication history

The pre-publication history for this paper can be accessed here:

http://www.biomedcentral.com/1472-6874/11/48/prepub

## References

[B1] Slieker-ten Hove MC Pool-Goudzwaard AL Eijkemans MJ Steegers-Theunissen RP Burger CW Vierhout ME Prediction model and prognostic index to estimate clinically relevant pelvic organ prolapse in a general female population Int Urogynecol J Pelvic Floor Dysfunct 2009 20 9 1013 21 10.1007/s00192-009-0903-0 19444367 PMC2721134

[B2] Patel DA Xu X Thomason AD Ransom SB Ivy JS DeLancey JO Childbirth and pelvic floor dysfunction: an epidemiologic approach to the assessment of prevention opportunities at delivery Am J Obstet Gynecol 2006 195 1 23 8 10.1016/j.ajog.2006.01.042 16579934 PMC1486798

[B3] Samuelsson EC Victor FT Tibblin G Svardsudd KF Signs of genital prolapse in a Swedish population of women 20 to 59 years of age and possible related factors Am J Obstet Gynecol 1999 180 2 Pt 1 299 305 9988790 10.1016/s0002-9378(99)70203-6

[B4] Wren PA Janz NK FitzGerald MP Barber MD Burgio KL Cundiff GW Optimism in women undergoing abdominal sacrocolpopexy for pelvic organ prolapse J Am Coll Surg 2008 207 2 240 5 10.1016/j.jamcollsurg.2008.02.019 18656053 PMC3709444

[B5] Boyles SH Weber AM Meyn L Procedures for pelvic organ prolapse in the United States, 1979-1997 Am J Obstet Gynecol 2003 188 1 108 15 10.1067/mob.2003.101 12548203

[B6] Olsen AL Smith VJ Bergstrom JO Colling JC Clark AL Epidemiology of surgically managed pelvic organ prolapse and urinary incontinence Obstet Gynecol 1997 89 4 501 6 10.1016/S0029-7844(97)00058-6 9083302

[B7] Fialkow MF Newton KM Lentz GM Weiss NS Lifetime risk of surgical management for pelvic organ prolapse or urinary incontinence Int Urogynecol J Pelvic Floor Dysfunct 2008 19 3 437 40 10.1007/s00192-007-0459-9 17896064

[B8] Subak LL Waetjen LE van den Eeden S Thom DH Vittinghoff E Brown JS Cost of pelvic organ prolapse surgery in the United States Obstet Gynecol 2001 98 4 646 51 10.1016/S0029-7844(01)01472-7 11576582

[B9] Subramanian D Szwarcensztein K Mauskopf JA Slack MC Rate, type, and cost of pelvic organ prolapse surgery in Germany, France, and England Eur J Obstet Gynecol Reprod Biol 2009 144 2 177 81 10.1016/j.ejogrb.2009.03.004 19414209

[B10] Barber MD Neubauer NL Klein-Olarte V Can we screen for pelvic organ prolapse without a physical examination in epidemiologic studies? Am J Obstet Gynecol 2006 195 4 942 8 10.1016/j.ajog.2006.02.050 16681989

[B11] MacLennan AH Taylor AW Wilson DH Wilson D The prevalence of pelvic floor disorders and their relationship to gender, age, parity and mode of delivery BJOG: An International Journal of Obstetrics & Gynaecology 2000 107 1460 1470 10.1111/j.1471-0528.2000.tb11669.x 11192101

[B12] Eva UF Gun W Preben K Prevalence of urinary and fecal incontinence and symptoms of genital prolapse in women Acta Obstet Gynecol Scand 2003 82 3 280 6 10.1034/j.1600-0412.2003.00103.x 12694126

[B13] Kumari S Walia I Singh A Self-reported uterine prolapse in a resettlement colony of north India J Midwifery Womens Health 2000 45 4 343 50 10.1016/S1526-9523(00)00033-7 10983434

[B14] World Health Organization. Measuring reproductive morbidity: report of technical working group Division of Family Planning 1989 (WHO/MCH/90.4)

[B15] Tegerstedt G Miedel A Maehle-Schmidt M Nyren O Hammarstrom M A short-form questionnaire identified genital organ prolapse J Clin Epidemiol 2005 58 1 41 6 10.1016/j.jclinepi.2004.06.008 15649669

[B16] da Silva GM Gurland B Sleemi A Levy G Posterior vaginal wall prolapse does not correlate with fecal symptoms or objective measures of anorectal function Am J Obstet Gynecol 2006 195 6 1742 7 10.1016/j.ajog.2006.07.034 17132476

[B17] Lukacz ES Lawrence JM Buckwalter JG Burchette RJ Nager CW Luber KM Epidemiology of prolapse and incontinence questionnaire: validation of a new epidemiologic survey Int Urogynecol J Pelvic Floor Dysfunct 2005 16 4 272 84 10.1007/s00192-005-1314-5 15856132

[B18] Romanzi LJ Chaikin DC Blaivas JG The effect of genital prolapse on voiding J Urol 1999 161 2 581 6 10.1016/S0022-5347(01)61957-8 9915453

[B19] Bradley CS Nygaard IE Vaginal wall descensus and pelvic floor symptoms in older women Obstet Gynecol 2005 106 4 759 66 10.1097/01.AOG.0000180183.03897.72 16199633

[B20] Burrows LJ Meyn LA Walters MD Weber AM Pelvic symptoms in women with pelvic organ prolapse Obstet Gynecol 2004 104 5 Pt 1 982 8 15516388 10.1097/01.AOG.0000142708.61298.be

[B21] Bump RC Mattiasson A Bo K Brubaker LP DeLancey JO Klarskov P The standardization of terminology of female pelvic organ prolapse and pelvic floor dysfunction Am J Obstet Gynecol 1996 175 1 10 7 10.1016/S0002-9378(96)70243-0 8694033

[B22] Chobanian AV Bakris GL Black HR Cushman WC Green LA Izzo JL Jr The Seventh Report of the Joint National Committee on Prevention, Detection, Evaluation, and Treatment of High Blood Pressure: the JNC 7 report JAMA 2003 289 19 2560 72 10.1001/jama.289.19.2560 12748199

[B23] Perkins NJ Schisterman EF The inconsistency of "optimal" cutpoints obtained using two criteria based on the receiver operating characteristic curve Am J Epidemiol 2006 163 7 670 5 10.1093/aje/kwj063 16410346 PMC1444894

[B24] Kestenbaum B Seliger SL Easterling TR Gillen DL Critchlow CW Stehman-Breen CO Cardiovascular and thromboembolic events following hypertensive pregnancy Am J Kidney Dis 2003 42 5 982 9 10.1016/j.ajkd.2003.07.001 14582042

[B25] Tan JS Lukacz ES Menefee SA Powell CR Nager CW Predictive value of prolapse symptoms: a large database study Int Urogynecol J Pelvic Floor Dysfunct 2005 16 3 203 9 discussion 209 10.1007/s00192-004-1243-8 15875236

[B26] Wang YP Teng CT Vieira Filho AH Gorenstein C Andrade LH Dimensionality of the premenstrual syndrome: confirmatory factor analysis of premenstrual dysphoric symptoms among college students Braz J Med Biol Res 2007 40 5 639 47 17464425 10.1590/s0100-879x2007000500006

[B27] Takeda T Tasaka K Sakata M Murata Y Prevalence of premenstrual syndrome and premenstrual dysphoric disorder in Japanese women Arch Womens Ment Health 2006 9 4 209 12 10.1007/s00737-006-0137-9 16761114

[B28] Indicators of coverage with primary health care services Analysis 2008 http://www.emro.who.int/dsaf/dsa1082.pdf,

[B29] Lee J The menopause: effects on the pelvic floor, symptoms and treatment options Nurs Times 2009 105 48 22 4 20050470

[B30] Singh K Reid WM Berger LA Assessment and grading of pelvic organ prolapse by use of dynamic magnetic resonance imaging Am J Obstet Gynecol 2001 185 1 71 7 10.1067/mob.2001.113876 11483907

